# Assessing the Diversity and Population Substructure of Sarda Breed Bucks by Using Mtdna and Y-Chromosome Markers

**DOI:** 10.3390/ani10122194

**Published:** 2020-11-24

**Authors:** Maria Luisa Dettori, Elena Petretto, Michele Pazzola, Oriol Vidal, Marcel Amills, Giuseppe Massimo Vacca

**Affiliations:** 1 Department of Veterinary Medicine, University of Sassari, via Vienna 2, 07100 Sassari, Italy; elenapetretto@outlook.it (E.P.); pazzola@uniss.it (M.P.); gmvacca@uniss.it (G.M.V.); 2 Departament de Biologia, Universitat de Girona, 17003 Girona, Spain; oriol.vidal@udg.cat; 3 Centre for Research in Agricultural Genomics (CRAG), CSIC-IRTA-UAB-UB, Department of Animal Genetics, Universitat Autònoma de Barcelona, 08193 Bellaterra, Spain; Marcel.Amills@uab.cat; 4 Departament de Ciència Animal i dels Aliments, Facultat de Veterinària, Universitat Autònoma de Barcelona, 08193 Bellaterra, Spain

**Keywords:** Y-chromosome, mitochondrial DNA, buck, population structuring, goat, SRY, AMELY, ZFY, DDX3Y

## Abstract

**Simple Summary:**

Domestic goats show extraordinary adaptation to different environments. Sardinia (Italy), the second-largest island in the Mediterranean Sea, is the birthplace of Sarda goats which are raised to produce milk mainly devoted to cheesemaking. The aim of this research was to characterize genetic diversity of Sarda bucks, reared in eight subregions of the island, to gain information about the relationship between genetic variation and geography as well as to investigate the existence of population substructure. We performed genotyping of Y-chromosome markers, which trace the patrilineal diversity, and sequencing of a portion of mitochondrial DNA, which gives information about matrilineal diversity. Analysis of Y-chromosome markers revealed the occurrence of high levels of diversity in populations from Southwest Sardinia. Further analysis of both Y-chromosome and mitochondrial DNA data evidenced the lack of population substructure. These results suggest the occurrence of extensive gene flow between the different subregions of Sardinia. The introduction of goats from other geographical locations, outside Sardinia, and belonging to highly productive breeds probably contributed to enhance genetic variation.

**Abstract:**

A sample of 146 Sarda bucks from eight subregions of Sardinia, Italy (Nuorese, Barbagia, Baronia, Ogliastra, Sarrabus, Guspinese, Iglesiente, Sulcis) were characterized for Y-chromosome and mtDNA markers to assess the levels of population substructure. Five polymorphic loci (SRY, AMELY, ZFY, and DDX3Y) on the Y-chromosome were genotyped. The control region of mtDNA was sequenced as a source of complementary information. Analysis of Y-chromosome data revealed the segregation of 5 haplotypes: Y1A (66.43%), Y2 (28.57%), Y1C (3.57%), Y1B1 (0.71%), and Y1B2 (0.71%). High levels of Y-chromosome diversity were observed in populations from Southwest Sardinia. The F_ST_ values based on Y-chromosome and mtDNA data were low, although a paternal genetic differentiation was observed when comparing the Nuorese and Barbagia populations (Central Sardinia) with the Sulcis, Iglesiente, and Sarrabus populations (Southern Sardinia). AMOVA analysis supported the lack of population substructure. These results suggest the occurrence of a historical and extensive gene flow between Sarda goat populations from different locations of Sardinia, despite the fact that this island is covered by several large mountain ranges. Introgression with foreign caprine breeds in order to improve milk production might have also contributed to avoiding the genetic differentiation amongst Sarda populations.

## 1. Introduction

Domestic goats (Capra hircus) are characterized by their extraordinary adaptation to different climates and environments. It is estimated that there are over a thousand goat breeds worldwide, with 579 local breeds officially reported by 182 countries from all over the world [1]. Most of these local breeds are reared under traditional low input extensive systems, particularly in developing countries [2]. In several regions of the Mediterranean basin, including Italy, dairy goat farming is practiced mainly in these traditional conditions. In Sardinia, an island off the west coast of Italy, dairy goat farming mainly takes place in the mountains and high hills of the Centre (Nuorese, Barbagia), East (Baronia, Ogliastra, Sarrabus), and South-West (Guspinese, Iglesiente, Sulcis) subregions of the island [3,4]. The autochthonous Sarda breed has a census of 260,000 heads, but only 6500 goats are registered in the herdbook [4,5]. Archaeological evidence suggests that domestic goats were present in Sardinia since the Neolithic age [6]. The main features of the Sarda goat breed, which is mainly devoted to cheese making [7], are a marked hardiness and striking morphologic (Figure S1) [4] and genetic [8,9,10] variability. In the recent past, Sarda goat keepers made unplanned crossings with exotic breeds (mainly Maltese), as a strategy to improve milk production [3,4]. This practice is considered detrimental for the conservation of the Sarda breed because uncontrolled admixture could lead to the dilution and erosion of its gene pool. In this regard, the FAO Committee on Genetic Resources in Agriculture [1] encourages a sustainable use of animal genetic resources in order to ensure that locally adapted breeds persist as functional components of production systems. One of the milestones to be achieved in order to safeguard the Sarda breed is to generate and gather information about its genetic variability.

The paternal history of breeds can be investigated with Y-chromosome markers, which have a male-limited transmission and do not recombine [11,12]. The diversity of the Y-chromosome has been reported for several goat breeds [13,14,15,16], demonstrating that the segregation of paternal markers is strongly associated with the continental origin of breeds. Other studies have used information enclosed in the mitochondrial DNA (mtDNA) to trace the matrilineal history of livestock species. In goats, research on mtDNA variability made it possible to detect a weak population structure that could be caused by both extensive gene flow and recent divergence [17,18,19,20]. Based on the analysis of the mtDNA hypervariable region of domestic goats, 6 highly divergent haplogroups (Hg) were described: A, B, C [17], D, F and G [18,21,22]. These Hg were already present 460,000 years ago in the wild ancestor of goats, the bezoar (*Capra aegagrus*), and their frequencies and geographic distribution reflect a population expansion event during the Neolithic transition that favored the worldwide spread of Hg A and, to a lower extent, of Hg C [23]. 

With regard to the Sarda goat breed, the diversity of the Y-chromosome was investigated in 190 bucks and it became evident that a Central European haplotype is particularly frequent, but the existence of population structure was not assessed [24]. Vidal et al. [16] also studied the Y-chromosome variation of Sarda bucks and demonstrated that Y1A is the most frequent haplotype followed by Y2. Three additional studies were conducted to characterize the variability of the mtDNA hypervariable region I (HVR-I) variability, but only in the female population [5,6,25]. In Sarda goats, the most frequent mtDNA haplogroup (Hg) is Hg A, but Hg C is also present [5,6]. In the context of the phylogenetic reconstruction carried out by Piras et al. [6], Hg A appears to be divided into 11 clades, and clade A11 is assumed to represent an ancient phylogenetic line. This line would be peculiar and exclusive of Sardinia, as later confirmed by Doro et al. [25].

The purpose of this research was to assess the patrilineal and matrilineal genetic diversity of a representative sample of Sarda bucks from different locations in order to find out whether variation patterns are associated with geography, a feature that was not assessed in previous studies. Moreover, we aimed to integrate both sources of information to investigate the existence of population substructure in the Sarda breed. Indeed, the bucks investigated in our study belong to the main subregions of Sardinia where the Sarda breed is reared, making it possible to determine whether mtDNA and Y-chromosome markers segregate differently depending on the geographic provenance of bucks. Population substructure can be produced by a broad array of causal factors including both neutral (genetic drift, founder effects, gene flow, etc.) and selective forces, so such information could provide valuable clues about the origin and history of the Sarda goat breed.

## 2. Materials and Methods 

### 2.1. Ethics Approval

For the present research, no specific authorization by an animal ethics committee was required. Blood samples were collected by private and official veterinarians of the local health authorities (ASSL) in the contest of sanitary programs, not linked with the present study.

### 2.2. Sampling and DNA Purification

A total of 146 bucks from 39 farms belonging to different subregions of Sardinia i.e., Nuorese (*n* = 12), Barbagia (*n* = 10), Baronia (*n* = 10), Ogliastra (*n* = 42), Sarrabus (*n* = 7), Guspinese (*n* = 10), Iglesiente (*n* = 15), Sulcis (*n* = 40), were sampled for this research. Sarda goats from these subregions differ in terms of farming methods and also for morphological and productive traits as a result of adaptation to different environmental conditions [3,4]. A general description of the sampled farms can be found in [5,26,27]. In each farm, one to five adult males were randomly chosen and blood samples were taken in K_3_EDTA vacuum tubes (BD Vacutainer, Becton Dickinson, Milan, Italy S.p.A.). Genomic DNA was isolated with the Gentra Puregene Blood Kit (Qiagen) and DNA purity and concentration were measured with an Eppendorf BioPhotometer instrument (Eppendorf). 

### 2.3. Y-Chromosome Genotyping

Seven single nucleotide polymorphisms (SNPs) located in the non-recombining region of the goat Y-chromosome were genotyped according to [16], i.e., SRY-2971 T > A, SRY-3098 G > A, SRY-1876 A > C (at the sex determining region Y gene), AMELY-42 C > T (amelogenin, Y-Linked, GenBank AY082491) and ZFY-527 A > G (zinc finger protein, Y-Linked, GenBank AY082500). These markers have been reported in previous studies [14,15,28,29], while markers DDX3Y g.56 C > G (DEAD-box helicase 3, Y-linked) and ZFY g.46 C > T were described for the first time by Vidal et al. [16]. A custom TaqMan Real-Time PCR assay was designed and genotypic data were generated on a 12K Flex QuantStudio instrument (Thermo Fisher Scientific) at the Servei Veterinari de Genetica Molecular from the Universitat Autònoma de Barcelona [30]. Genotypes were visualized with the Taqman Genotyper v.1.3 software (Applied Biosystems) and SNPs with call rates < 0.9 were removed from the dataset.

### 2.4. Mitochondrial DNA Analysis

The control region of the mtDNA was amplified with the primer pair MT1F: 5′-AGCCATAGCCTCACTATCAGC-3′ and MT1R: 5′-TCATCTAGGCATTTTCAGTG-3′ [31] to yield a fragment of 1316 bp (including the complete control region of mitochondrial DNA) by using PCR conditions described by [31]. Amplified products were purified with ExoSAP (Applied Biosystems): 10.7 µL amplified product were mixed with 4.3 µL of ExoSAP reagent and incubated at 37 °C for 15 min on a thermal cycler. Subsequently, ExoSap was inactivated at 80°C for 15 min and the purified amplified product was used as a template to carry out sequencing reactions with the BigDye Terminator v3.1 Cycle Sequencing Kit (Applied Biosystems). Sequencing reactions were run on an ABI PRISM 3730 automated sequencer (Applied Biosystems). In cases where a full length sequence was not obtained, we used the primer pairs MTAF: 5′-CGCTCGCCTACACACAAATA-3′ and MTAR: 5′-AATGCCCATGCCTACCATTA-3′ [32] to generate the missing sequence data. For mtDNA sequences, the software Phred/Phrap/Crossmatch were used to compute data quality information from chromatogram files [33,34]. PolyPhred was used to compare sequence traces and search for polymorphisms within the aligned sequences [35,36]. Then, Consed was used to view polymorphism tags by navigating through the sequence traces in order to visually check all chromatogram peaks [37,38,39]. 

### 2.5. Statistical and Computational Analysis

The software MEGA version 7.0 [40] was used to align mtDNA sequences. Indel and missing data were removed from the alignment before any analysis was performed. The DnaSP v.5.10.01 software [41] was used to calculate nucleotide and haplotype diversities and the F_ST_ values according to [42]. The software Network v.10 [43] was employed to build Median-Joining (MJ) networks based on Y-chromosome and mtDNA data. Polymorphic sites were weighted inversely to the number of mutational events needed to solve the network, as in [31]. Transversions and transitions were given weights of 3 and 1, respectively. Analysis of molecular variance (AMOVA) was carried out with the Arlequin 3.5 software [44] with default parameters. The POPART v.1.7 software [45] was used to display Y-chromosome haplotype frequencies for each of the geographical locations where samples were collected. A Neighbour Joining (NJ) tree based on F_ST_ values (for Y-chromosome data) was built with the MEGA 7.0 software.

## 3. Results

### 3.1. Y-Chromosome Markers

Of all animals tested for Y-chromosome markers, 140 bucks from 38 farms were correctly genotyped. Out of the 7 SNPs under study, one was monomorphic: AMELY-42 C > T (GenBank AY082491). The remaining SNPs defined five haplotypes (Table 1). Haplotype frequency and diversity in each subregion are described in Table 2. The value of overall haplotype diversity was 0.479. At the subregional scale, the Sarrabus population was highly diverse despite its low sample size, with an Hd value (0.571) higher than those observed in the Sulcis (0.529) and Ogliastra (0.504) populations, which are represented by more individuals. Nuorese (Hd = 0.166), Barbagia (Hd = 0.200), and Baronia (Hd = 0.250) populations displayed the lowest Hd values, and Iglesiente (0.670) was the most diverse Sarda population. 

The estimates obtained for F_ST_ coefficients are described in Table 3. The F_ST_ values among populations ranged between 0.00 and 0.16. Most populations displayed little genetic differentiation (F_ST_ < 0.05), with a few exceptions, e.g., Barbagia and Nuorese vs. Sulcis (F_ST_ 0.12 and 0.14, respectively), Sarrabus (F_ST_ 0.13 and 0.16, respectively) and Ogliastra (F_ST_ 0.13 and 0.16, respectively).

Performance of an AMOVA analysis demonstrated that the between-populations component of variation is very small (2.47%) and that population structure was not significant (Table 4).

**Figure 1:**
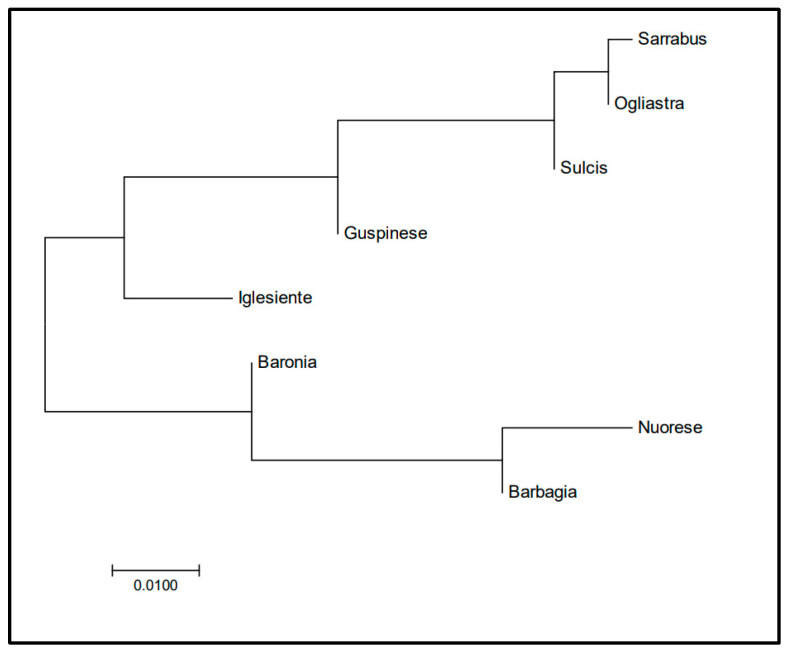
Neighbour joining tree of Sarda bucks from different Sardinia locations based on F_ST_ coefficients estimated from Y-chromosome data. Populations included in the phylogenetic tree are: Nuorese (*n* = 12), Barbagia (*n* = 10), Baronia (*n* = 10), Ogliastra (*n* = 42), Sarrabus (*n* = 7), Guspinese (*n* = 10), Iglesiente (*n* = 15), Sulcis (*n* = 40).

The NJ tree based on F_ST_ data (Figure 1) showed that three populations (Nuorese, Barbagia, and Baronia) are differentiated from the remaining ones.

**Figure 2:**
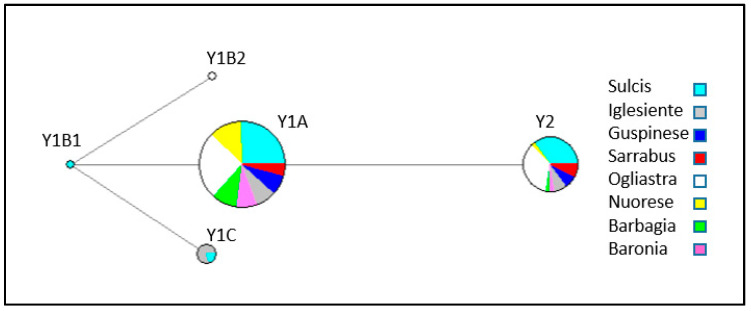
Median joining network based on Y-chromosome haplotypes of 140 Sardinian bucks.

The MJ network based on Y-chromosome haplotypes (Figure 2) contained two Y1A and Y2 clades with contributions from all eight Sarda populations. In addition, haplotype Y1C was harboured by individuals from Iglesiente and Sulcis, while haplotypes Y1B1 and Y1B2 only segregated in Sulcis and Ogliastra individuals, respectively.

**Figure 3:**
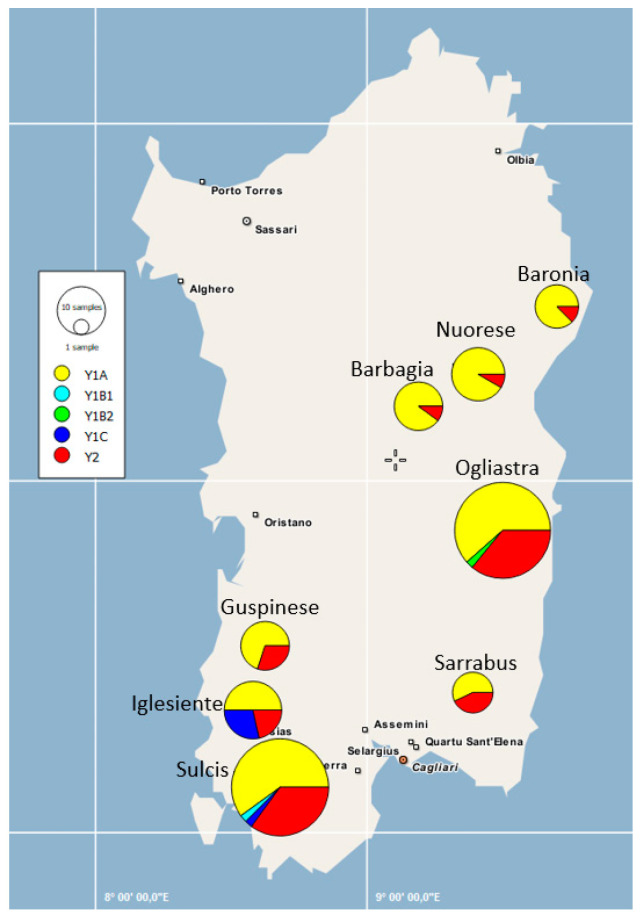
Sampling area and geographic distribution of the Y-chromosome haplotypes of Sarda bucks in the island of Sardinia (Italy). The size of each circle is proportional to the number of individuals.

Haplotype clustering based on the geographical coordinates of sampled locations are depicted in Figure 3. It can be observed that the three populations from Nuorese, Barbagia, and Baronia differed from the remaining ones with regard to the frequency of haplotype Y1A.

### 3.2. Mitochondrial DNA Analysis

Sequencing of the hypervariable region of mtDNA was achieved for 146 bucks. Taking as a reference sequence NC_005044.2 [46], most of the samples were sequenced from nucleotide (nt) 15437 to nt 16600. All non-redundant sequences were submitted to GenBank (Accession Numbers MN786532-MN786639). For the downstream analyses, two sequences were excluded from the dataset (MN786581 and MN786585) because they contained 76 and 77 bp insertions, respectively. The overall alignment of 925 bp highlighted the existence of 120 variable sites and 94 haplotypes. Moreover, haplotype diversity was 0.992 (Table 5). The highest haplotype diversity value was observed in the Barbagia population, with 10 different haplotypes out of 10 sampled animals (Hd = 1). 

**Figure 4:**
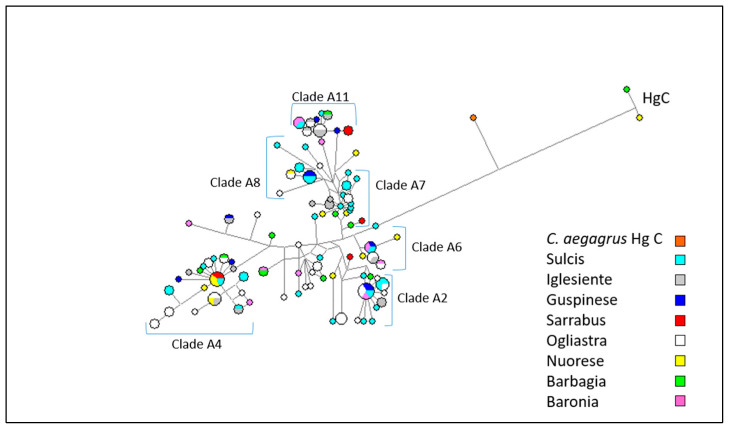
Median joining network based on mtDNA haplotypes of 144 Sardinian bucks. The size of each circle is proportional to the number of individuals.

A MJ network including 144 mtDNA sequences from Sarda bucks (from nt 15437 to nt 16361 of NC_005044.2) is shown in Figure 4. The outgroup is represented by a mitochondrial sequence from a Capra aegagrus (KR059221) individual carrying haplogroup C. 

According to the phylogenetic reconstruction carried out by [6], Sarda goat haplotypes are distributed over 11 Hg A clades. In order to understand which clades our sequences belong to, we combined our dataset with that of [6] and we performed a haplotype analysis using the DnaSP software v.5.10.01 [41]. As shown in Table S1, clades A4 (23), A11 (19), and A2 (18) comprised of the highest proportion of Sarda buck haplotypes. No haplotype fell into clade A9, while 26 haplotypes did not match any haplotype of the Sarda goat dataset reported by [6]. The MJ network based on mtDNA data showed some haplotype clusters that were indistinctly contributed by the eight populations, thus indicating the absence of genetic differentiation between populations. Some haplotype clusters were attributable to clades A2, A4, A6, A7, A8, and A11 previously identified in Sarda goats [6]. The two haplotypes belonging to Hg C were present in Barbagia and Nuorese bucks. 

**Figure 5:**
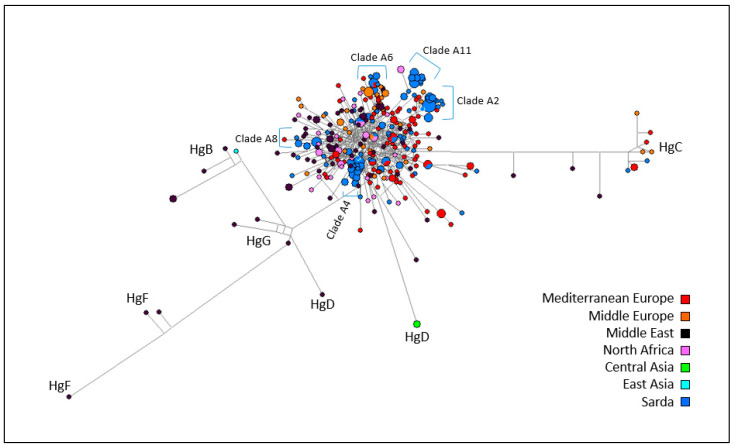
Median joining network based on mtDNA sequences from goats from Middle Europe (37), Mediterranean Europe (87), Middle East (73), North Africa (32), and 144 Sardinian bucks. The size of each circle is proportional to the number of individuals.

A second MJ network including all mtDNA sequences from Sarda bucks plus European, Asian, and African caprine mtDNA sequences retrieved from the public databases and representing known haplogroups (File S1) was also built (Figure 5). In the resulting MJ network, the Sarda buck haplotypes were distributed over the entire Hg A cluster. It was possible to detect several haplotypes matching the clades defined by [6]. Clade A11 was clearly separated from the remaining Hg A sequences, including one haplotype of African origin. Clades A6 and A2 also showed genetic differentiation, and included, in addition to the Sarda buck haplotypes, haplotypes of goats from Middle Europe, Mediterranean Europe (clade A2) and Middle East (clade A6). Clades A8 and A4 were also recognizable within Hg A. 

The measurement of F_ST_ values amongst populations based on mtDNA haplotypes yielded negative or near zero values, indicating the absence of population substructure The AMOVA analysis confirmed the absence of population substructure (Table 6).

## 4. Discussion

### 4.1. Patterns of Y-Chromosome and Mitochondrial Diversity in Sarda Bucks 

Analysis of Y-chromosome variation at SRY, AMELY, ZFY, and DDX3Y genes in 140 Sarda breed bucks revealed the segregation of all the haplotypes identified so far in Europe, Africa, and the Near East [16]. Sechi et al. [24] analyzed eight SNPs in the Y-chromosome of Sardinian bucks, demonstrating that paternal genetic diversity is high. Vidal et al. [16] also investigated Y-chromosome variation in the Sarda breed bucks and showed that the frequencies of the Y1A, Y1B2, and Y2 haplotypes were approximately 72%, 5%, and 23%. However, the experimental design of the two studies mentioned before hindered the performance of analyses of genetic diversity at the regional scale or population substructure at the within-breed level. 

In the present investigation, we have been able to compare the genetic variation of Sarda bucks from different geographic areas. Indeed, we have observed very different values of Hd among the eight populations under consideration. With regard to Y-chromosome variation, the higher haplotype diversity was found in populations from the South-West (Iglesiente, Sulcis, Guspinese) and in Sarrabus and Ogliastra. The MJ network (Figure 1) showed that the two main haplotypes were Y1A and Y2, and these two haplotypes segregated in all populations under analysis. In contrast, the Y1B1, Y1B2, and Y1C haplotypes had low frequencies and were detected only in Ogliastra (Y1B2), Sulcis (Y1B1 and Y1C), and Iglesiente (Y1C). Ogliastra and Sulcis populations are represented by the largest number of sampled animals, so the presence of multiple haplotypes could be expected to some extent. However, the Iglesiente population was only represented by 14 bucks and, despite this, it displayed the highest haplotypic diversity observed in our data set (Hd = 0.67). 

These observations are consistent with the fact that farmers from the Southwestern region of Sardinia have often imported bucks from exotic breeds in order to improve milk production [47]. Indeed, haplotypes Y1B2, Y1B1, and Y1C are particularly frequent in Swiss breeds [16]. In contrast, goat farmers from Nuorese and Barbagia (but also Baronia and Ogliastra) have tended to preserve goat herds from the introgression of foreign breeds, a course of action that is probably explained by the orographic, cultural, and historical particularities of this territory [48]. Possibly, the introgression of Sarda goats with exotic breeds had a different impact on the paternal and maternal gene pools, because the analysis of mtDNA data showed patterns that are very different from those inferred from Y-chromosome data. Indeed, the Sulcis population was the one with the lowest nucleotide diversity and Hd values, while these two parameters reached high values in the case of the Nuorese and Barbagia populations. Differential rates of foreign introgression for females and males might explain the discrepancy observed between mtDNA and Y-chromosome data, but this should be further tested with autosomal markers in order to reach a valid conclusion.

The analysis of mtDNA data also highlighted that the majority of Sarda mtDNA haplotypes belong to the A haplogroup, which is strongly predominant in all goat breeds. Interestingly, HgC, which has scattered distribution throughout Europe, was also present in Sarda bucks. This haplogroup is supposed to derive from a second wave of domestication, more recent than Hg A [6]. Noteworthy, 13.2% of the bucks analyzed belonged to HgA clade A11, which is considered to be specific to Sardinia. This clade could be traced back to an ancient line the origins of which have not been defined yet, so its segregation cannot be attributed to the importation of goats from Europe [25].

### 4.2. Lack of Population Substructure in the Sarda Breed

In general, the F_ST_ values measured on the basis of Y-chromosome data were quite low (F_ST_ < 0.05), but two populations (Barbagia and Nuorese) displayed an increased level of differentiation with regard to buck populations from Sarrabus, Sulcis, and Ogliastra. These differences can be better visualized by examining the geographic distribution of Y-chromosome haplotypes in Sarda bucks (Figure 3). Importantly, the AMOVA analysis reported in Table 4 made evident the absence of a significant paternal population substructure (*p*-value = 0.18) in the Sarda breed. Data obtained from the mtDNA sequences from 144 Sarda bucks confirmed these findings (Figure 4), which were further supported by the fact that all F_ST_ coefficients amongst populations were close to zero. This lack of population substructure suggests the occurrence of extensive gene flow between goat populations from different geographic areas of Sardinia. In this island, it is common for sheep farmers to practice inverse transhumance in which permanent settlements are established in the mountains and flocks travel down to the lowlands for winter [49]. This practice is not common for goat herds reared with traditional extensive methods, which are allowed to graze very large and uncontrolled territories, up to thousand hectares, rented by farmers from the municipalities [7]. In summary, the high mobility of Sarda goats might have facilitated the occurrence of genetic exchanges between herds from different locations of Sardinia. It is important to emphasize, however, that the analysis of autosomal genome-wide SNP markers might allow to discern levels of population substructure, in the Sarda breed, that are not detectable with mitochondrial and Y-chromosome markers. Indeed, the genotyping of caprine populations with the Goat SNP50 BeadChip has made possible to dissect population structure and genetic differentiation much more efficiently than the analysis of mitochondrial sequences [17,50]. Thus, we cannot rule out the possibility that the mitochondrial and Y-chromosome markers used in the current work do not have enough resolution to capture fine levels of population structure in the Sarda breed.

The occurrence of multiple imports of animals along different historical periods since the Neolithic age might have also contributed to weakening of genetic differences between different Sarda populations. Cardoso et al. [51] analysed the patterns of homozygosity in continental and insular goat populations and concluded that insular populations from Madagascar and Iceland and La Palma showed increased levels of homozygosity when compared to nearby continental populations, while such observation was not true for Sarda goats. Throughout history, Sardinia has been an important port-of-call of maritime routes that were navigated by Phoenicians, Carthaginians, Greeks, Romans, Arabs, and many other seafaring civilizations [52]. This circumstance probably enhanced the frequent admixture of caprine populations with different gene pools. Furthermore, a recent mixed ancestry, with a major influence of Maltese goats, has been revealed for the Sarda breed [53]. 

## 5. Conclusions

We have examined the variation of Y-chromosome and mtDNA markers in bucks from different geographic areas of Sardinia. Our results indicate the absence of population substructure, with very low F_ST_ values amongst populations and non-significant results in the AMOVA analysis for both paternal and maternal markers. These results are compatible with the occurrence of an extensive gene flow between goat populations raised in different locations from Sardinia, despite the fact that this island has a rugged landscape and is covered by large mountain ranges. Moreover, the practice of introducing allochthonous breeds to increase milk production of Sarda goats might have contributed to weaken genetic differentiation amongst populations. We have also observed a higher Y-chromosome haplotype diversity in bucks from Southwestern Sardinia, but such pattern was not detected when analyzing mtDNA markers. This result highlights that the paternal and maternal histories of the Sarda breed are not fully concordant, but the causes of these differences remain to be fully elucidated. From a conservation perspective, our study indicates that the Sarda breed, in contrast with other local populations from Southern Europe [31], has not suffered a strong reduction of its genetic diversity, probably because of the existence of a substantial gene flow amongst different populations distributed across Sardinia. Of more concern would be the potential introgression of Sarda goats with foreign breeds, a feature that could dilute the gene pool of the Sarda breed and decrease its adaptation to current breeding conditions in Sardinia. In this regard, it would be critical to make sure that uncontrolled crossbreeding between Sarda goats and exotic breeds does not take place since this is essential to preserve this unique genetic resource.

## Figures and Tables

**Table 1:** Y-chromosome polymorphisms and haplotypes analyzed in 140 bucks from the Sarda breed.

Haplotype	SRY 2971	SRY 3098	SRY 1876	AMELY 42	ZFY 527	ZFY 46	DDX3Y 56	Haplotype Frequency%
Y1A	T	G	A	C	A	C	G	66.44
Y2	T	A	A	C	G	C	C	28.57
Y1C	A	G	C	C	A	C	G	3.57
Y1B1	A	G	A	C	A	C	G	0.71
Y1B2	A	G	A	C	A	T	G	0.71

**Table 2:** Distribution of Y-chromosome haplotypes in Sarda bucks from eight regions of Sardinia.

Region	Number of Haplotypes	Haplotype Diversity	Number of Animals
Nuorese	2	0.166	12
Barbagia	2	0.200	10
Baronia	2	0.250	8
Ogliastra	3	0.504	39
Sarrabus	2	0.571	7
Guspinese	2	0.466	10
Iglesiente	3	0.670	14
Sulcis	4	0.529	40
Overall population	5	0.479	140

**Table 3:** F_ST_ values calculated with DnaSP from Y-chromosome data.

Heading	Sulcis	Guspinese	Sarrabus	Ogliastra	Nuorese	Baronia	Barbagia
Guspinese	0.00						
Sarrabus	0.00	0.00					
Ogliastra	0.00	0.00	0.00				
Nuorese	0.14	0.05	0.16	0.16			
Baronia	0.07	0.00	0.08	0.08	0.00		
Barbagia	0.12	0.02	0.13	0.13	0.00	0.00	
Iglesiente	0.05	0.03	0.05	0.06	0.09	0.05	0.07

**Table 4:** Analysis of molecular variance (AMOVA) based on Y-chromosome data from Sarda bucks distributed in eight regions of Sardinia.

Source of Variation	d.f.	Sum of Squares	Variance Components	Percentage of Variation
Among populations	7	6.828	0.01755 Va	2.47%
Within populations	132	91.350	0.69205 Vb	97.53%
Total	139	98.179	0.70960	
Fixation Index F_ST_: 0.02473		*p*-value = 0.18		

**Table 5:** Nucleotide and haplotype diversities of 144 Sarda bucks based on mtDNA sequence data.

Region	Nucleotide Diversity, Π	Number of Haplotypes	Haplotype Diversity, Hd	Sample Size
Nuorese	0.017	11	0.984	12
Barbagia	0.020	10	1.000	10
Baronia	0.015	9	0.977	10
Ogliastra	0.013	30	0.984	42
Sarrabus	0.012	4	0.866	6
Guspinese	0.013	8	0.955	10
Iglesiente	0.013	13	0.980	15
Sulcis	0.012	32	0.989	39
Overall population	0.013	94	0.992	144

**Table 6:** Analysis of molecular variance (AMOVA) based on mtDNA data of Sarda bucks from eight subregions of Sardinia.

Source of Variation	d.f.	Sum of Squares	Variance Components	Percentage of Variation
Among populations	7	40.850	−0.02873 Va	−0.46%
Within populations	136	858.553	6.31289 Vb	100.46%
Total	143	899.403	6.28416	
Fixation Index F_ST_: −0.00457		*p*-value = 0.62		

## Supplementary Materials

The following are available online at https://www.mdpi.com/2076-2615/10/12/2194/s1, Figure S1: Bucks and goats of Sarda breed, File S1: List of mtDNA Sequences. Table S1: Distribution of the bucks mtDNA haplotypes in the 11 clades representing haplogroup A.
