# Supplementary material for: Assessing the Diversity and Population Substructure of Sarda Breed Bucks by Using Mtdna and Y-Chromosome Markers
Source: Animals (Basel). 2020 Nov 24;10(12):2194. doi: 10.3390/ani10122194 (PMC7761473; doi:10.3390/ani10122194)
Supplement: Supplementary file 1 [file animals-10-02194-s001.zip › Supplementrary -animals-1006852/Supplementary Figure 1.pdf]

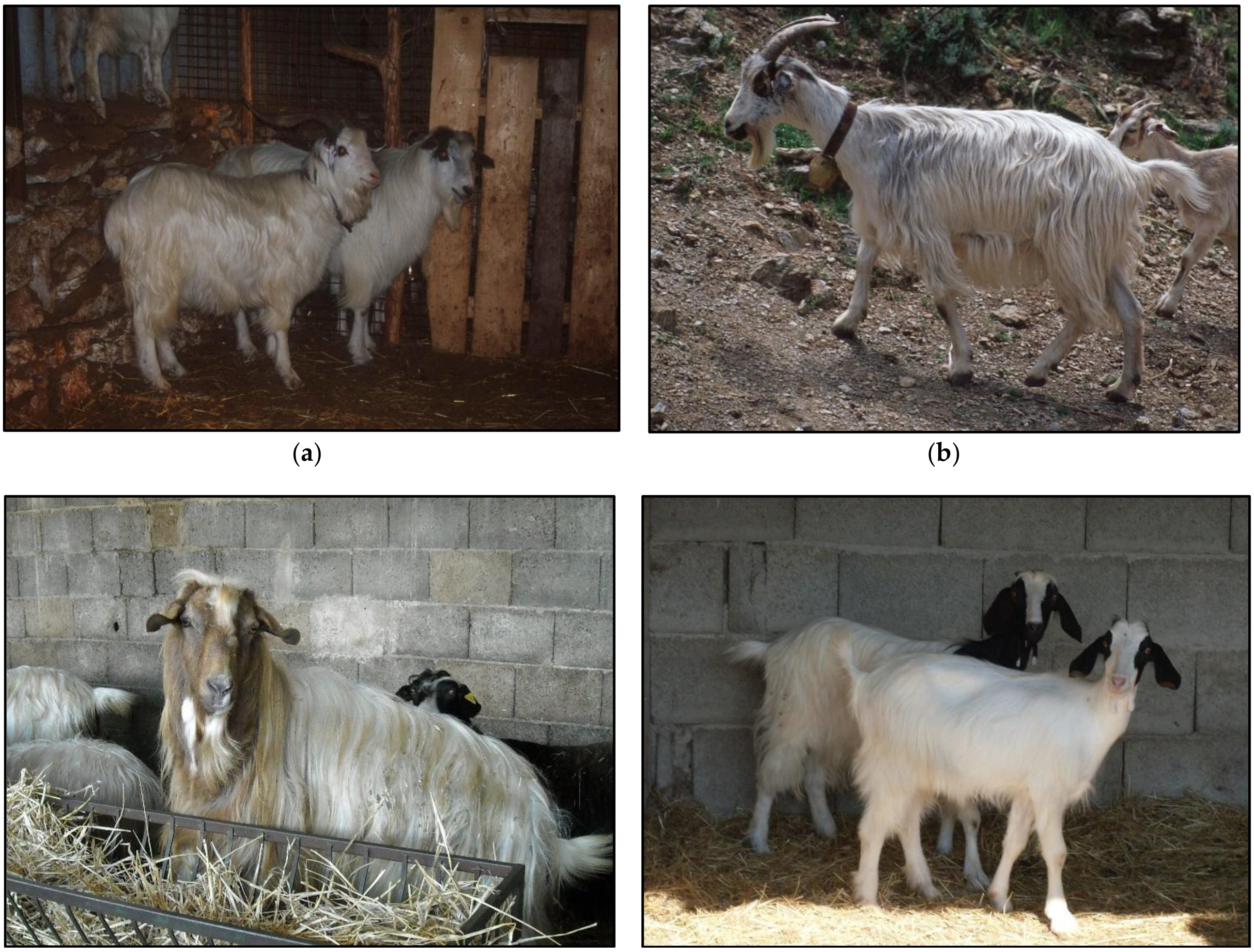

(**a**) (**b**) (**c**) (**d**)

Supplementary Figure 1. Bucks and goats of Sarda breed from the subregions of Sardinia Nuorese, Barbagia, Baronia, Ogliastra and Sarrabus, showing the common morphological traits of the ancient breed: short ears, horned, grey coat (**a, b**); and from the subregions Guspinese, Iglesiente and Sulcis, showing the morphological traits of imported breeds: long ears, polled, black or red patched coat (**c, d**). Original pictures by G.M. Vacca and M. Pazzola.
