# Supplementary material for: Assessing the Diversity and Population Substructure of Sarda Breed Bucks by Using Mtdna and Y-Chromosome Markers
Source: Animals (Basel). 2020 Nov 24;10(12):2194. doi: 10.3390/ani10122194 (PMC7761473; doi:10.3390/ani10122194)
Supplement: Supplementary file 1 [file animals-10-02194-s001.zip › Supplementrary -animals-1006852/Supplementary Table 1.pdf]

**Supplementary Table 1**. Distribution of the bucks mtDNA haplotypes in the 11 clades representing haplogroup A according to Piras et al., (2012).

| Clade | Region | Number of Haplotypes |
|---|---|---|
| A1 | Nuorese, Sulcis | 2 |
| A2 | Barbagia, Baronia, Ogliastra, Guspinese, Iglesiente, Sulcis | 18 |
| A3 | Ogliastra, Sarrabus | 4 |
| A4 | Nuorese, Barbagia, Baronia, Ogliastra, Sarrabus, Iglesiente, Sulcis | 23 |
| A5 | Barbagia, Baronia, Ogliastra, Guspinese, Iglesiente, Sulcis | 11 |
| A6 | Baronia, Ogliastra, Guspinese, Iglesiente, Sulcis | 6 |
| A7 | Barbagia, Baronia, Ogliastra, Iglesiente, Sulcis | 11 |
| A8 | Nuorese, Ogliastra, Guspinese, Sulcis, | 10 |
| A9 | - | 0 |
| A10 | Ogliastra, Sulcis | 2 |
| A11 | Barbagia, Baronia, Ogliastra, Sarrabus, Guspinese, Iglesiente, Sulcis | 19 |
